# Obstetrics

**Published:** 1878-05

**Authors:** 


### Obstetrics.

Laparo-Elytrotomy.—Dr. T. G. Thomas. (*Am. Jour. of Obstetrics*, Apr., 1878.)

Dr. Thomas devotes some space to a historical sketch of the surgical procedures resorted to in order to save the life of the mother or child when delivery *per vias naturales* is impossible, considering the dangers, feasibility, and humanity of each. He discards statistics of such operations entirely, inasmuch as the successful cases only are usually reported.

With regard to the operation which he makes the subject of his paper, he says that since he fully elaborated his own idea of it, his attention was called to the fact that a very similar method was employed in one case by Ritgen, who suggested it in 1820, as a modification of one proposed by Jörg. To Dr. T., however, is due the credit of having independently conceived it, of having proved its practicability by operation upon the cadaver—the subject of the experiment having been the body of a woman who died in the ninth month of her pregnancy; and finally of having twice performed the operation with results not so easily attainable in any other way.

His first case was that of a woman *in articulo mortis* (result of pneumonia), where the chief object was to save the child if possible, and at the same time render the mother's closing hours free from the agony of labor. This result was readily accomplished; but the child proved to be premature and deformed, and died about the same time with the mother.

He then records three cases by Dr. Skene, of Brooklyn—one where craniotomy had already been done, and where the mother survived the operation some hours, finally dying of exhaustion from her prolonged labor, and two where both mother and child were saved. A fifth case (Dr. T.'s second) was also thus doubly successful; thus making a total of three mothers saved, and four children delivered alive out of the five cases; a remarkable record.

Comparing his own operation with the others alluded to in the introductory part of the paper, he finds that the dangers of metritis, peritonitis, and incarceration of the intestines in a uterine rent, are entirely obviated, while those of shock and septicæmia are very much diminished. There is a possibility of cellulitis, and three of the above cases were complicated by fistulous openings in the bladder, which proved, however, a comparatively trivial matter, and partly the result of insufficient experience. The great danger is that of hemorrhage. This has not yet been encountered, but there is reason to fear it.

With regard to the details of the operation, and points chiefly to be observed, they are briefly these: the operator should be provided with pocket case, anæsthetic, Barnes' dilators, and some actual cautery appliance. The patient, fully anæsthetized, must be placed upon a firm table, and the os fully dilated by appropriate measures. An incision is then made from the anterior superior spine of one ilium to the spine of the pubis, just above and parallel to Poupart's ligament, and carried through the muscles and soft parts to the peritoneum; this will be found ample and movable, and must be lifted up and back. A sound or other guide in the vagina is then cut down upon, the vaginal wound sufficiently enlarged, and the child delivered by version, if an arm or the head present, by extraction if the breech do so. Then, the placenta having been delivered, the uterus is caused to fully contract, suitable measures are taken for stopping hemorrhage, and interrupted silk sutures introduced to close the abdominal wound. Observance of the usual precautions familiar to all surgeons, will, in all probability, insure a happy result.

THE TWINS OF ST. BENOIT. (*L'Union Médicale du Canada.*)

We visited, a few days ago, the freak of nature which has for a short time past been on exhibition to the Montreal public. This consists of a peculiar modification of twins—two heads, necks and thoraces, and four arms; one abdomen, and two legs. The trunks blend together in the most curious way. The upper part of the trunks are quite distinct, but at the juncture of the thoraces with the abdomen, the two become united, so that whilst it is evident that there are two thoraces, there appeared to be but one abdominal cavity. The angle formed by the division above the abdomen is 125°, that is the thoracic axes are at an angle of about 60° with the abdominal axis. From the pelvis down, the parts appear quite natural. At the first glance, the abdomen, which has only one navel, seems to manifest nothing more than an unusual development. But on more attentive examination, it is evident that the intestines are quite distinct for each trunk. This is manifest, because when one side coughs, makes a long inspiration, cries, or defecates, the abdominal agitation is only visible on one side, while the other side remains tranquil. Moreover, when certain movements are made, a slight ridge in the median line is manifest through the whole abdomen, which indicates the division of the separate intestines. But what is remarkable, is that there is only one anus and one vagina, while the acts of defecation and micturition are independent for each side of the abdomen.

The legs—only two—are not associated with the same nervous system. This becomes evident by an observation of the two when only one is asleep.

The delivery was effected without difficulty; indeed a physician was not called. The mother is well and strong, and the progeny promises to live long. The babies are nine weeks old, in good health, and very pretty. The left is a little more plump than the right, but the general health and strength appear to be alike.

Chloral in Retention of Urine.—In the *Gazetta Medica de Roma*, Dr. Tidd narrates a case of the retention of urine relieved by chloral hydrate. A young woman in the eighth month of pregnancy, believed herself in labor, and had not passed her urine for twenty-four hours. The bladder was greatly distended, and projected prominently above the pubes and into the vagina; so much so that it was impossible to reach the neck of the uterus with the finger. The external organs of generation were excessively tumefied and the suffering great. Morphia was administered, and for fear of rupture of the bladder, puncture was proposed. The proposition was rejected, and the following administered :

℞ Chloral. hydrat. .............................. ʒ ijj

Aquæ ........................................ ℥ ij m.

Sig. One teaspoonful every half hour—later, every two hours.

Deep sleep soon followed, and five minutes after the administration of the second dose the evacuation of urine commenced, the patient being unconscious. Seven days afterwards normal delivery; no further retention.

Subcutaneous Injections of Ergotine in Metrorrhagia.—(*Journ. de Médecine*, Nov. 1877.)

M. Constantine Paul has made an interesting communication on this subject to the Société de Thérapeutique, reproduced by the *Bulletin général de Thérapeutique.*

The solution he uses, is that of Moutard-Martin, to-wit: water 15 grammes, glycerine 15 grammes, ergotine 2 grammes.

In thirteen cases of metrorrhagia following cancers, or abortions, Paul has seen the hemorrhage cease in from five to ten minutes. In each case, 66 milligrammes of ergotine were injected, that is to say a gramme of the solution.

The advantages of this method are incontestable, and far superior to the use of ergot by the stomach, for several reasons; first, the rapidity with which it acts, a fact of extreme importance when the hemorrhage menaces the life of the patient. Besides, the colic caused by the injection of ergot, is avoided, and finally the effect produced is of longer duration.

Transfusion.—Dr. M'Clintock, of the Dublin Obstetrical Society. (*Dublin Jour. of Med. Sci.*, March, 1878.)

A patient was nearly exsanguinated by flooding during labor. When Dr. M. reached her bedside, he found her in a most alarming condition, insensible and almost pulseless. Examination revealed a leg in the vagina. He at once delivered the child, which had evidently been dead but a short time. Up to the time of his visit, she had been in labor five hours. In spite of every precaution and effort, including the hypodermic use of sulphuric ether, she did not rally—the surface being cold and clammy, and the pulse barely perceptible. Transfusion offering the only hope, he sent for Dr. McDonnel, who brought with him the apparatus bearing his name. Nine hours after delivery, the operation was performed, ℥x of blood being taken from the husband and defibrinated, and then slowly injected into the right arm. Immediate improvement in the pulse was noticed, and in a few hours there were most encouraging indications of reaction. Convalescence was perfectly satisfactory.

The reporter alluded to an equally successful case of Dr. Beatty's, the only difference being that, in the latter case, an interval of fourteen hours elapsed between delivery and transfusion. Thus ample time was given in each case for reaction to assert itself, if it would. Dr. M'Clintock considers that these two cases offer very conclusive evidence of the value of transfusion under similar circumstances and as a *dernier resort.*

